# The high-dimensional geographic dataset revealed significant differences in the migration ability of cadmium from various sources in paddy fields

**DOI:** 10.1038/s41598-023-28812-9

**Published:** 2023-01-28

**Authors:** Feng Wang, Yanqiu Zhang, Ting Wu, Lina Wu, Guoliang Shi, Yi An

**Affiliations:** 1 Agro-Environmental Protection Institute, Ministry of Agriculture and Rural Affairs, Tianjin, 300071 China; 2 College of Environmental Science and Engineering, Nankai University, Tianjin, 300350 China; 3 College of Resource and Environment, Huazhong Agricultural University, Wuhan, 430070 China; 4 State Key Laboratory on Odor Pollution Control, Tianjin Academy of Eco-Environmental Sciences, Tianjin, 300191 China

**Keywords:** Environmental sciences, Environmental impact

## Abstract

Cadmium (Cd) contamination in paddy fields and its subsequent transfer in soil–rice systems are of particular concern. Significant discrepancies exist in the transfer process of Cd pollution sources from soil to rice. Here, we proposed a novel hybrid framework to reveal the priority of controlling Cd pollution sources in soil–rice systems, based on a high-dimensional geographical database. We further defined transfer potential (TP) to describe the ability of Cd from soil to rice (TP^r^ = Cd_r_/Cd_s_) and activated status (TP^a^ = Cd_a_/Cd_s_), respectively, to reveal the priority sources of Cd pollution at the regional scale. The mining source has both high levels of TP^r^ and TP^a^, which should be a controlled priority. Followed by traffic sources with a higher value of TP^r^, showing the risk to rice rather than the soil. The activated and enriched capacities of soil Cd are unequal in different sources that we attribute to the disparities of Cd transport in soil–rice systems. Cd contamination shows a significant spatial heterogeneity due to the difference in its transport performance. Our findings provide support for designing site-specific and pollution-targeted control priorities for suitable Cd pollution mitigation strategies at the regional scale.

## Introduction

Rice is a leading staple food for over half of the world’s population. China's rice intake (214.19 g/capita/day) has exceeded the world average (148 g/capita/day) by 45%^1^. Especially the Yangtze River basin supplies the highest amount of commercial rice in China due to the abundant water, light, and heat resources^1,2^. With the rapid development of industrialization and the intensification of agricultural activities, heavy metal pollution of agricultural soil and its subsequent transfer to the rice chain has attracted worldwide concern, which has a severe impact on soil ecosystem function and food security^3–5^. Heavy metals accumulated in agricultural soils could result in the degeneration of soil biology and function, and then posed an adverse effect on human health through the food chain^6,7^, such as arsenic (As), cadmium (Cd), and lead (Pb) could damage neurocognitive ability and vascular complications, and even cause cancer^8,9^.

In recent decades, there have been many events of cadmium contamination of rice in the Yangtze River basin, especially in Hunan, which have posed public health risks^10,11^. Cd, one of the most toxic heavy metals in agricultural soils, has been reported as the most serious heavy metal contamination of agricultural land^12,13^. Cd is a non-essential element for plant growth, however, it is easily absorbed by the roots of crops because of its high mobility and potential bioavailability among soil elements. Rice is more available to accumulate Cd compared with other cereals, making it a significant source of human Cd risk^14,15^. Thus, it is important to learn about the sources of Cd pollution that may protect human food safety.

Currently, research on the source appointment of Cd pollution in paddy soil–rice systems has received a boost from the growing concern about soil ecosystem function and food security. Zou et al.^1^ summarized the significant natural and anthropogenic sources in China, including mining activities, intensive application of phosphates fertilizers, and e-waste. In addition, sewage irrigation and acid mine drainage are also sources of cadmium in the paddy soil–rice system^16,17^. The aforementioned studies focus on soil Cd contamination rather than the Cd in rice. While the Cd transport and accumulation from soil to rice are complex. Physicochemical characteristics of soil, soil microorganisms, and the physiological features of rice plants contributed to Cd accumulation in rice grains, which can be controlled to mitigate activated cadmium and rice cadmium^1,14^. However, rarely studies were conducted on the source appointment of activated cadmium and rice cadmium. Therefore, it is essential to reveal the pollution of activated cadmium and rice cadmium to achieve Cd mitigation.

The conventional cadmium source appointment methods include the isotope natural abundance ratio method and pollutant discharge inventory method^18–20^. However, the former method is not suitable for regional-scale analysis due to its high detection cost and complex interaction among pollutant sources. While the latter is also limited to lacking human pollution activity data and accurate statistics of emissions from various sources. It is also difficult to analyze the cadmium transformation process. Recently, receptor models have been gradually carried out in source apportionment of Cd pollution in soil, such as principal component analysis/multiple linear regression (PCA/MLR)^4,21^ and positive matrix factorization (PMF)^6,22^. Although receptor models have successfully been used to identify the contribution of pollution sources to Cd in the soils, rarely have investigations focused on sources of activated cadmium and rice cadmium. Therefore, the identification and control of pollution sources in soil and rice should be differentiated and targeted.

Paddy plants have been considered strong Cd pollution accumulators^23^. Cd pollution paddy soil–rice systems and its successive accumulation in the rice plant have been studied in many research. In the Cd uptake and accumulation mechanism in the paddy soil–rice system, due to the significant differences in Cd absorption and translocation process, there is a huge difference between Cd contamination in soil and rice^1,19,24^. For instance, the reduction of the physicochemical contents (carbonate and sulfide) of soil can increase the concentration of the Cd in soil^7^. In addition, previous studies focused on the source of Cd in soil and the input of total Cd pollution^16,25^. However, due to the difference in Cd activity in soil and rice, controlling the soil Cd pollution source may not be effective in reducing Cd concentration in rice. Priority should be given to the sources with the highest activity rather than those with the highest total input. To reduce Cd concentration in rice effectively, it is necessary to consider the differences in Cd activity of different pollution sources in the soil–rice system comprehensively.

Xiangtan County, Hunan Province, is China’s commercial grain production place, of which 80% of farmland is used for growing rice^2^. However, a previous study has reported that the average Cd content of rice exceeded the standard by 88%, and rice consumption accounted for 81% of Cd intake^1,2^. Hence, we took Xiangtan County as the case of a typical Cd pollution area and proposed a novel hybrid framework to reveal the priority of controlling Cd pollution sources in the paddy soil–rice system. In the framework, the local industry types of enterprises are classified; then local Cd input flux estimation and PCA-MLR are combined to explore the Cd pollution sources. Finally, we defined transfer potential (TP) to express the ability of Cd from soil to rice (TP^r^ = Cd_r_/Cd_s_) and activated status (TP^a^ = Cd_a_/Cd_s_) and revealed the priority of pollution sources.

## Materials and methods

### Study area

**Figure 1:**
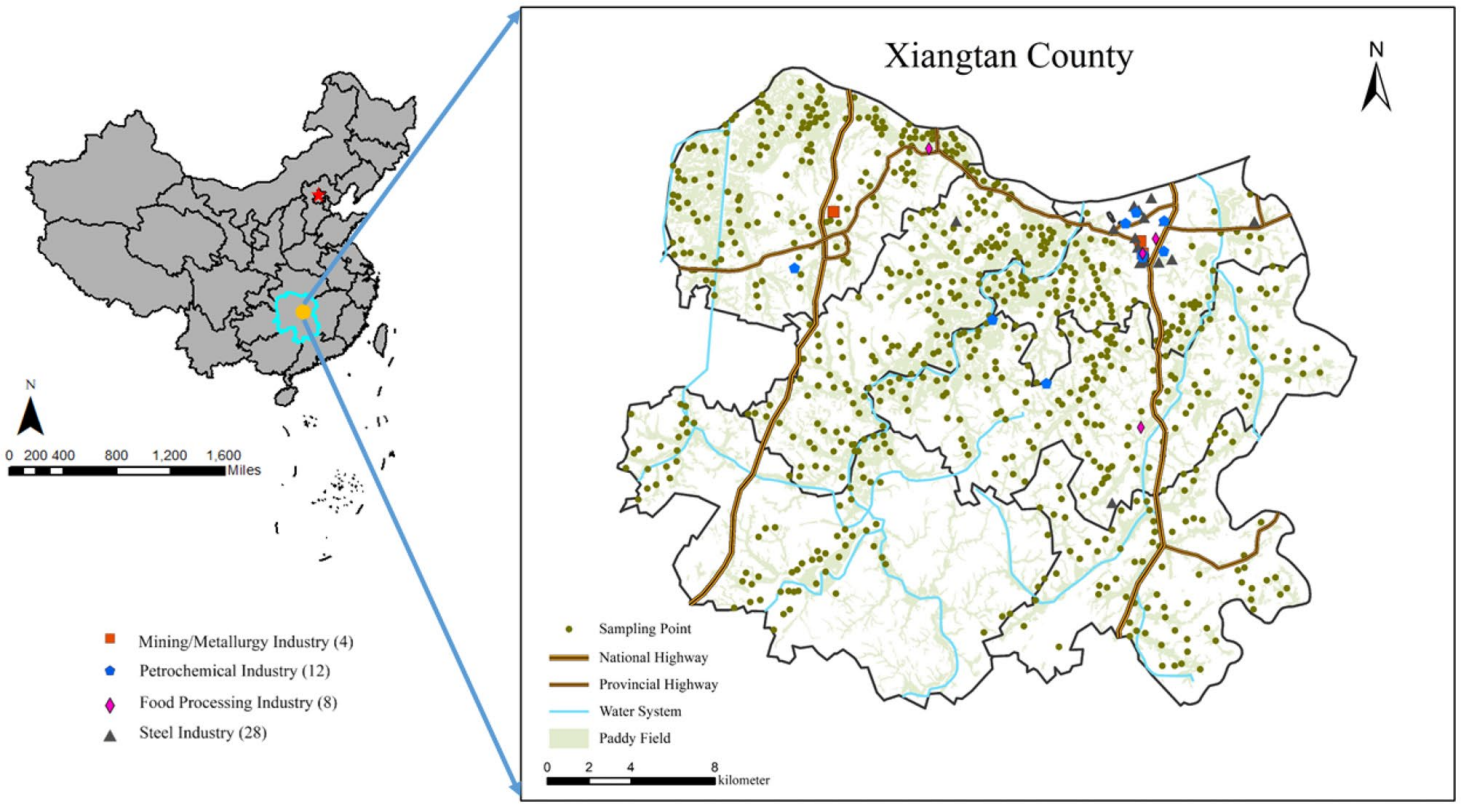
Spatial distribution of monitoring sites in the study area. Pictures were generated by ArcGIS software 10.3; ArcGIS Online.

This research is carried out in Xiangtan County (E112°25′–113°03′, N27°20′–28°05′), Hunan Province in China (Fig. 1). It is lying in Central China and is close to the China East Sea with a subtropical monsoon climate zone. The annual mean temperature and rainfall are 17.5 °C and 1300 mm, respectively. The study area is located in the Yangtze River basin which is characterized by abundant water and light. Therefore, it has been the major rice-producing area partly due to policy support, market demand, and agricultural development^1^. However, the cadmium pollution of the paddy soil–rice system in this region was serious as the exploitation of non-ferrous metal mines and the irrigation pollution of the Xiangjiang River. In addition, the large population, dense traffic network, and high investment in agricultural production have further exacerbated cadmium pollution^26^. Hence, we select six towns with typical characteristics of serious cadmium pollution^27^ as the case areas in this study.

### Soil and rice sampling and laboratory analysis

A field sampling campaign was conducted during the rice harvest period, in which a total of 733 pairs of topsoil samples (0–20 cm) and rice samples were collected (Fig. 1). The rice samples complied with relevant institutional, national, and international guidelines and legislation, such as Procedural regulations regarding monitoring of pollutants in the produces of agriculture, animal husbandry and fishery [NY/T 398-2000]. The sampling sites were recorded as a global positioning system (GPS) and randomly arranged, while the sampling density was proportional to the over-standard rate of cadmium in the town, ranging from 2.13 to 6.16 sites/km^2^. Each sampling site was far away from roads, fields, compost heaps, etc. The sampling sites of rice are corresponding to those of soil samples one by one. All the soil and rice samples were stored and then immediately transported to the laboratory. The plant material used in our study complied with relevant institutional, national, and international guidelines and legislation, such as Procedural regulations regarding monitoring of pollutants in the produces of agriculture, animal husbandry, and fishery [NY/T 398-2000]^28^.

Approximately 0.100–0.250 g of soil sample was weighed and then digested in the microwave digester with the mixed acid solution of HNO_3_–HF–HClO_4_ (volume ratio 1:2:1). In terms of rice sample, a 0.250 g sample was taken into the digestion tank with HNO_3_–HClO_4_ (volume ration 9:1), and then digested in the microwave digester after standing for about 12 h. All soil–rice samples were prepared and analyzed by national standards or industrial standards. The monitoring indexes and their corresponding detection methods were listed in Table S1. The soil cadmium (Cd_s_) and rice cadmium (Cd_r_) were the concentrations of Cd in soil and rice, respectively. The activated cadmium (Cd_a_) was defined as the concentrations of Cd in soil extracted by CaCl_2_ [DB35/T 860-2008]. For dataset quality control, we replaced element measurements greater than 3 standard deviations with the mean value of it. The monitoring results of soil properties in the study area were provided in Table S2.

### High-dimensional geographical database

**Figure 2:**
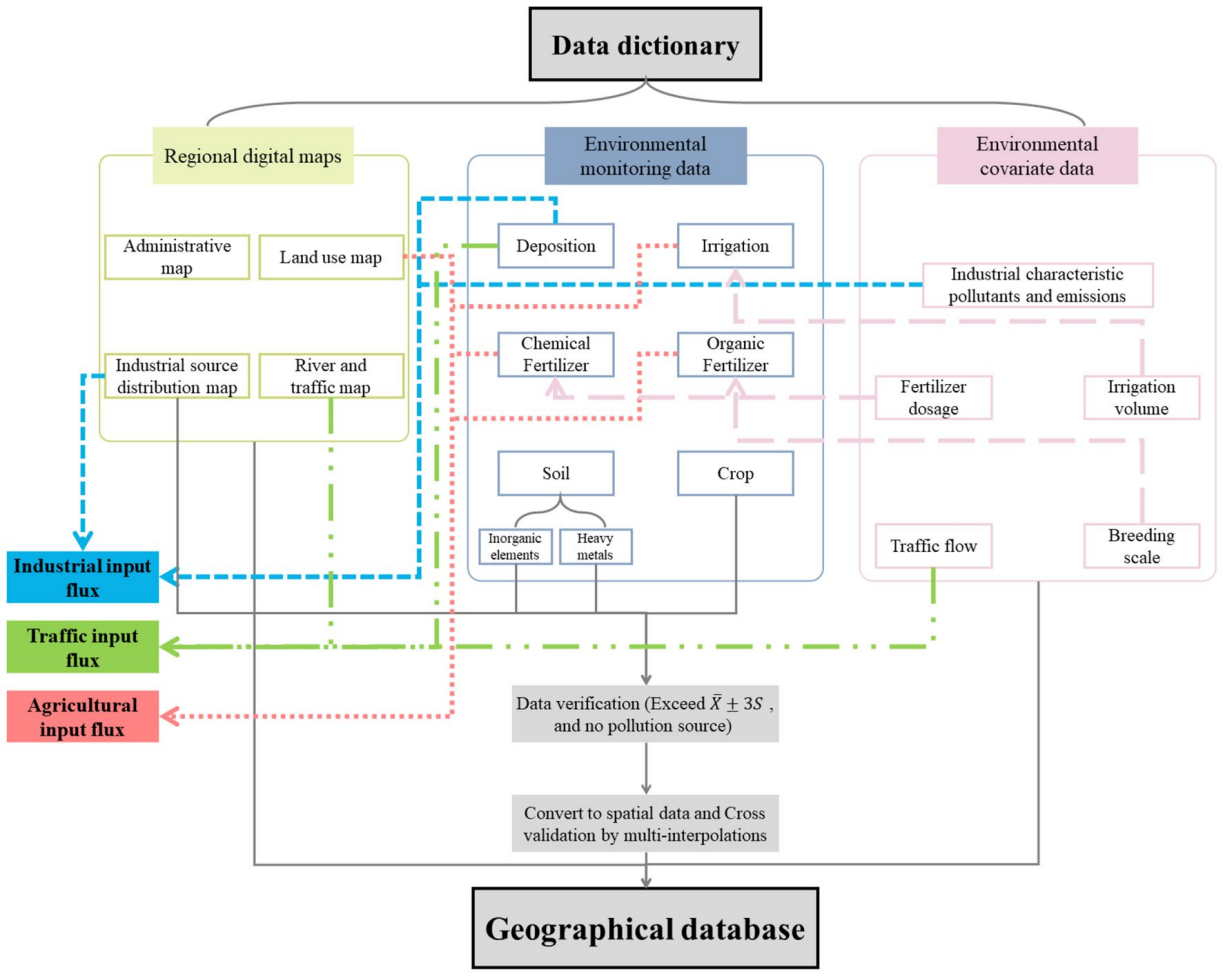
The high-dimensional geographical database.

The high-dimensional geographical database consists of three parts: regional digital maps, environmental monitoring data, and environmental covariate data (Fig. 2). Thereinto, regional digital maps contain four parts: administrative map, land use map, industrial source distribution map, and river and traffic map. Environmental monitoring data contain six parts: deposition, irrigation, chemical fertilizer, organic fertilizer, soil, and crop. Soil data contains inorganic elements and heavy metals. Environmental covariate data contain five parts: industrial characteristic pollutants and emissions, fertilizer dosage, irrigation volume, traffic flow, and breeding scale. An industrial source distribution map, deposition, and industrial characteristic pollutants and emissions are calculated by industrial input flux. River and traffic maps, deposition, and traffic flow are related to traffic input flux. Land use maps, irrigation, chemical fertilizer, and organic fertilizer are connected to agricultural input flux. In addition, fertilizer dosage, irrigation volume, and breeding scale are in connection with chemical fertilizer, irrigation, and organic fertilizer, respectively. The data of soil, crop, and industrial source distribution are converted to spatial data after data verification, and then the cross-validation was conducted by multi-interpolations.

### The preparation and validation of Kriging interpolation methods

The Kriging interpolation methods and their key parameters were given in Table S3. The kriging method was used to map the spatial distribution of element concentrations. Cd, As, Cr, Hg, Cu, P, N, Na, Mo, Mn, Mg, Ca, Al, and Ni are best fit with simple Kriging method, Pb, Zn, K, Si, Se, Fe, and Ti are best fit with common kriging method. Among Kriging interpolation method parameters, Nugget/Sill ratio can be traditionally regarded as the criterion to classify the spatial dependence of soil properties^24^. For instance, Nugget/Sill ratio > 75%, 25–75%, and < 25% represent the weak, moderate, and strong spatial autocorrelation, respectively. Cd, As, Cr, Zn, Cu et al. had strong spatial dependence. The accuracy of the interpolation was validated utilizing root mean square error (RMSE). The RMSE values were all close to 1 and less than 2, suggesting the interpolation in this study was acceptable.

### The hybrid framework

**Figure 3:**
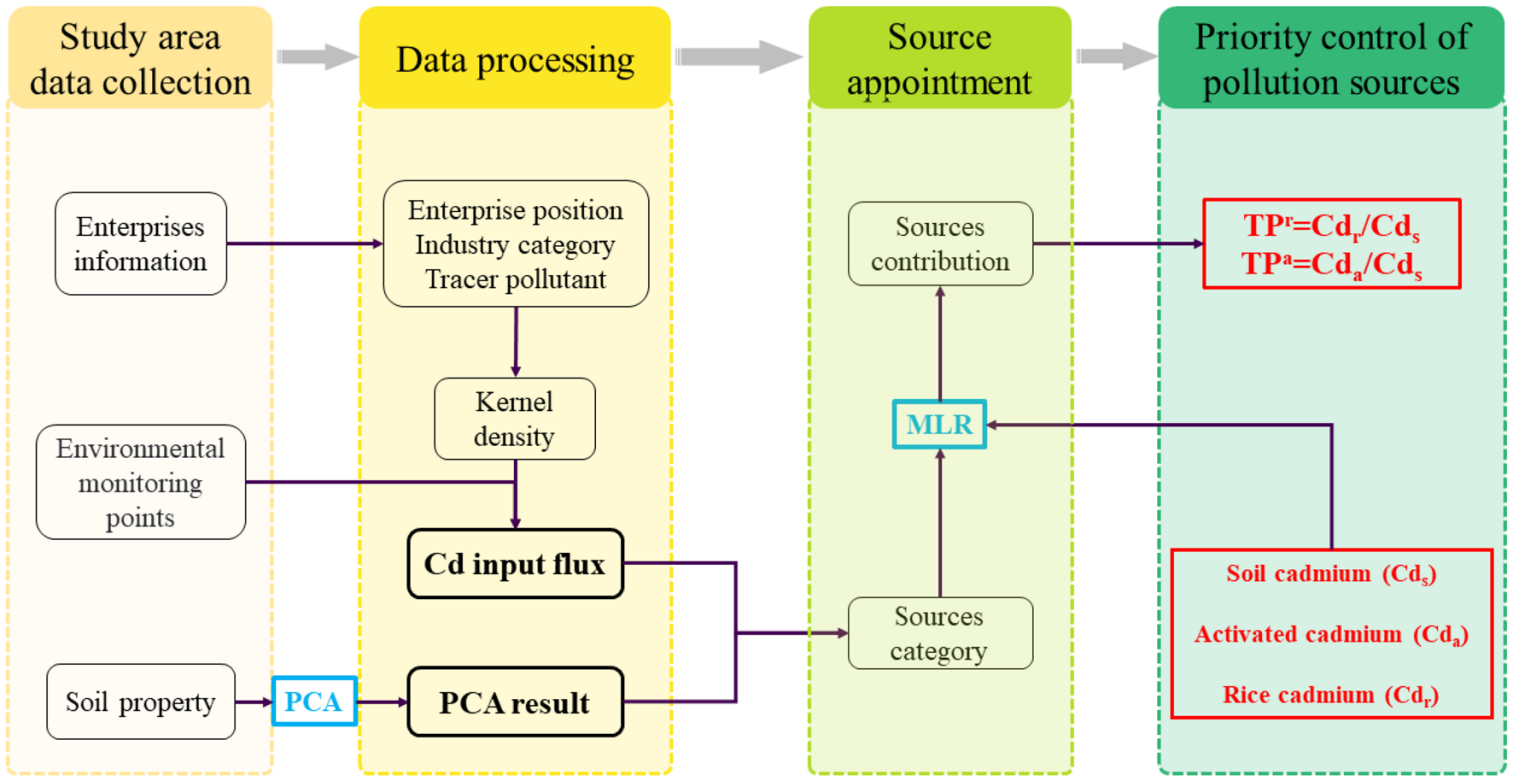
Workflow of the proposed hybrid framework for revealing the Cd pollution sources for priority control in soil–rice systems factors.

Here, we proposed a novel hybrid framework to reveal the priority of controlling Cd pollution sources in soil–rice systems (Fig. 3). The main process are as follows.

1. Enterprises information collection and classification

We collect the enterprise names, industry categories, and business scopes in the study area on the web crawler from China Organization Data Service (https://www.cods.org.cn), to optimize the classification of business categories. The industry category standard is based on the text of “Industrial Classification for National Economic Activities” [GB/T 4754-2017]. Therefore, we extract four types of business categories: the mining and smelting industry, the petrochemical industry, the food processing industry, and the steel industry.

(2) Cd Contaminating enterprise density to estimate Cd input flux

We carried on the kernel density (KD) estimation of the aforementioned four Cd contaminating enterprise distribution by ArcGIS 10.6^29^. Therefore, the Cd input flux is estimated:

1 $$Cd\;Flux = \mathop \sum \limits_{k = 1}^{4} \left[ {\frac{{KD_{k} - KD_{\min } }}{{KD_{\max } - KD_{\min } }}/\left( {k \times \left( {Flux_{Atmosphere} + Flux_{Rainfull} } \right)} \right)} \right]$$

2 $$Flux_{Atmosphere} = Cd \times Flux_{dust}$$

3 $$Flux_{Rainfull} = Cd \times annual\;precipitation$$

where *k* is the number of the types of business categories, $KD_{k}$ is the lattice logarithmic kernel density, $KD_{\min }$ is the minimum logarithmic kernel density, $KD_{\max }$ is the maximum logarithmic kernel density, $Flux_{Atmosphere}$ is the Cd atmospheric flux at the sampling point, $Flux_{Rainfull}$ is the Cd rainfall flux at the sampling point. In addition, we also calculated the Cd fluxes of livestock (cattle, sheep, pigs, and poultry) and fertilizer utilizing (N, P, K, and compound fertilizers) in the study area.

(3) PCA-MLR model

The PCA-MLR model used in pollution source appointment started in atmospheric particulate matter^30^ and has been broadened to the source analysis of soil and groundwater^31–33^. Then, least squares regression^34^ was used to calculate the principal component scores in all monitoring sites. First, the pollution source analysis is realized by PCA as the following equation:

4 $$C_{ij} = \mathop \sum \limits_{k} S_{ik} L_{kj}$$

where *i*, *j*, and *k* are the sample, pollutant specie, and pollution source, respectively; *C*_*ij*_ is the standardized normal concentration of all pollutant species. *S*_*ik*_ is the synthetic contribution of the *k* th source to the *i* th sample (score matrix), *L*_*kj*_ is the fraction of the *j* th species in *k* th source.

Then, MLR was applied to the score matrix to determine the contribution percentage of Cd to each pollution source. As shown in Eq. (5), the concentration of Cd was set as the dependent variable and the absolute principal component score (*S*_*ik*_) was set as the independent variable:

5 $$Cd_{i} = \mathop \sum \limits_{k} S_{ik} A_{k}$$

where Cd_*i*_ is the standardized concentration of Cd in all samples, *A*_*k*_ is the coefficient of the regression for the *k* th pollution source. Finally, the contribution ratio of the soil, activated and rice Cd contents (Cd_s_, Cd_a_ and Cd_r_) to different sources were calculated:

6 $${\upomega }_{{\text{k}}} = \frac{{A_{k} }}{{\mathop \sum \nolimits_{k} A_{k} }}$$

where ω_k_ was the contribution rate of a certain source.

## Results and discussion

### Cd contamination in paddy soil–rice system

**Figure 4:**
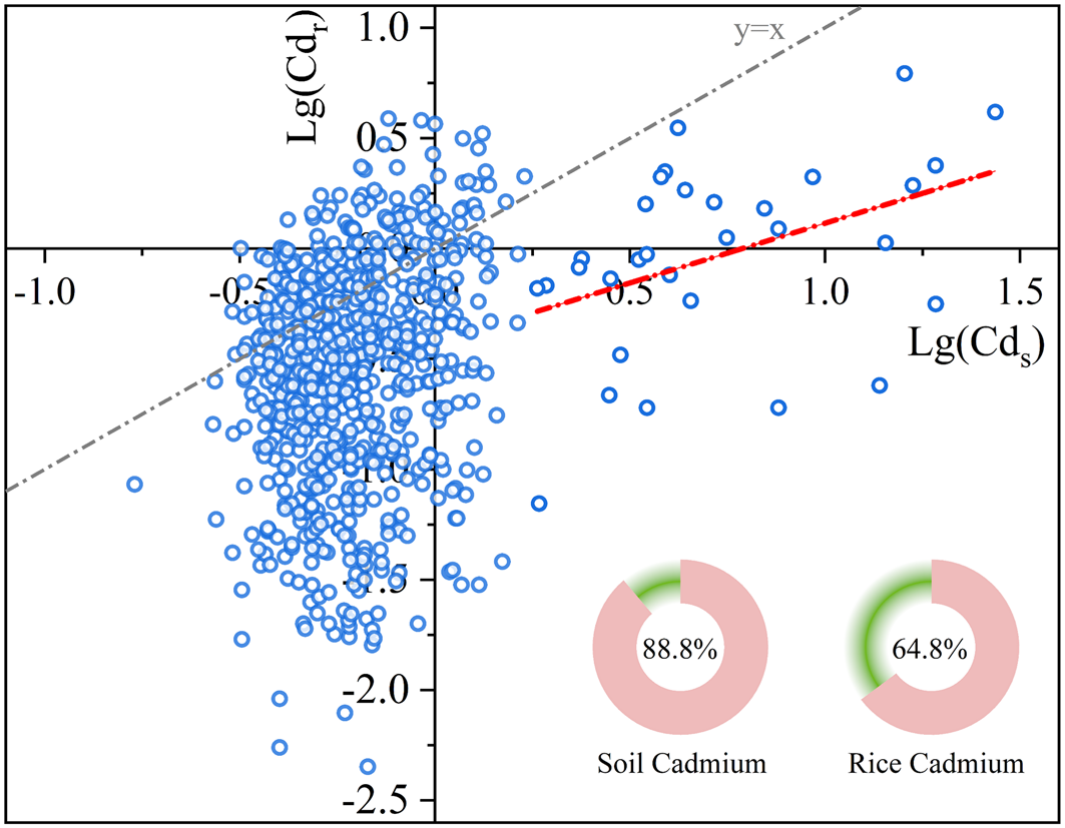
The cadmium contents in soil and rice in the study area. The pie chart showed the exceeding standard rates of cadmium in soil and rice, respectively.

Table 1 summarizes the descriptive statistics of soil cadmium (Cd_s_), activated cadmium (Cd_a_), and rice cadmium (Cd_r_) in soil–rice systems. The average Cd concentrations in soil and rice are 1.11 and 0.52 mg/kg, respectively. Both of them exceed the national standard limits (GB 15618-2018, GB2762-2017) by 88.8 and 64.8% (Fig. 4). In terms of Cd_a_, the average concentration is 0.63 mg/kg exceeding the Cd_r_, suggesting the severe food risk of rice. We caution that the Cd_a_/Cd_s_, as the bioavailability of Cd in soils, are exceedingly high levels which reached 90.1%. This implies that the Cd_a_ plays a more crucial role than Cd_s_ in the accumulation of Cd in rice. Our results are in line with a previous study that Cd accumulation is often controlled by its bioavailability than its total content in the soil to a greater extent^14,35^.

**Table 1:** The monitoring results of soil cadmium, activated cadmium, and rice cadmium.

	Min	Max	Mean	Median	Over standard rate (%)
Cd_s_ (mg/kg)	0.17	27.3	1.11	0.60	88.80
Cd_r_ (mg/kg)	0.01	4.15	0.52	0.33	64.80
Cd_a_ (mg/kg)	0.23	5.36	0.63	0.53	–
Cd_a_/Cd_s_	0.036	3.335	0.910	0.851	–

Figure 4 illustrates the cadmium contents in soils (Cd_s_) and rice (Cd_r_) based on paired samples of soil and paddy rice at the sampling site. The concentration of Cd_s_ does not correlate with Cd_r_ the contents of Cd in soils were up to 1.5 mg/kg, and its concentrations in rice grain were generally below 0.75 mg/kg. This implied that the elevated Cd_s_ levels do not necessarily result in excessive accumulation of Cd_r_. While lg(Cd_r_) increases approximately linearly with lg(Cd_s_) exceeding 0.25, up to 1.5. This suggests that when Cd concentrations are higher in soil, it is more likely of Cd transfers to rice. Many factors affected the uptake of Cd by rice roots and translocation and accumulation in grains, such as soil pH, redox potential, and clay minerals^14^. Many regression models have been conducted on the relationship between rice cadmium and soil properties as above^24,36^. However, the ability of rice to absorb and accumulate Cd can differ significantly due to the soil conditions and management practices at spatial and temporal scales^14^. As a result, the nonlinear relationship of Cd_s_ transferred to Cd_a_ and Cd_r_ is detrimental to designing suitable Cd mitigation strategies in soil–rice systems. Revealing the pollution sources of soil cadmium, activated cadmium, and rice cadmium in the soil–rice system may benefit Cd control.

### Source appointment

**Figure 5:**
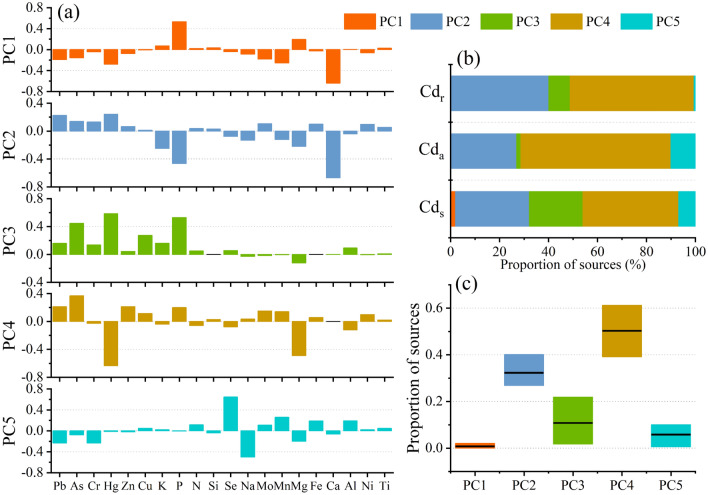
The results of PCA, (**a**) the component matrix of the most important five principal components, (**b**) the contribution ratios for different sources, and (**c**) the mean and range of the contribution ratios of different sources.

To reveal the significant differences in the migration ability of cadmium, we applied PCA-MLR to identify the specific sources of soil cadmium, activated cadmium, and rice cadmium in the soil–rice system. Five factors were identified as shown in Fig. 5a. Factor 1 (PC1) was characterized by high levels of element P, and a positive correlation with K. As we know, phosphate fertilizer and potassium fertilizer are indispensable for the growth of rice^37,38^. Therefore, PC1 was assigned to farming sources, especially phosphate fertilizer. Factor 2 (PC2) was correlated strongly with Pb and Zn. Previous studies have reported that brakes, tires, and lubricating oil contains considerable Pb and Zn^39,40^ that are easily released into the ambient and then deposited into the soils. Some studies have confirmed that Pb and Zn are the trace element of traffic emissions^41,42^. Therefore, PC2 was considered as traffic emissions. Factor 3 (PC3) was dominated by high proportions of As, Hg, and Cu. Traditionally, As and Hg were regarded as an indicator of agricultural activities, including fertilizers, pesticides, and herbicides^24,43,44^. Moreover, As and Cu are usually used as feed additives in livestock diets^6,45^. Hence, As, Hg and Cu were at high levels in livestock and poultry manure and can be migrated into the soil during manure applications, as previously reported suggested^46^. So, PC3 is identified as an animal husbandry source. Factor 4 (PC4) was strongly enriched with many heavy elements, such As, Zn, Pb, Hg, etc., which might be associated with industrial activities^47^. Previous studies revealed that heavy metal elements mainly originated from industrial emission such as gold smelting, metal ore mining, smelting, and processing activities, and acid mine drainage^48,49^. In our study area, many mining industries and chemical enterprises are all around the main roads and rivers (Fig. 1). Therefore, F4 was regarded as a mining source. The last Factor 5 (PC5) should be geological sources attributing to the indication of Se, Mn, Fe, and Al^25,50^ which are regarded as the soil parent materials. In addition, based on the estimation of Cd input flux in the study area, it is found that there are six main sources of Cd pollution (Fig. S1): mining and smelting industry, petrochemical industry, food processing industry, steel industry, animal husbandry, and fertilizer use. Thereinto, the mining and smelting industry are mainly phosphate rock mining, and the petrochemical industry is related to traffic emissions. Therefore, we grouped the mining and smelting industry and fertilizer use as farming sources. In summary, Cd input flux is mainly from five main sources which are in line with PCA results.

The identified five factors have a cumulative contribution rate of more than 85% and the contribution of each pollution source was calculated as shown in Fig. 5b. The mining source is the dominant contributor among the five sources, which contributed 50.3 ± 11.1% (Fig. 5c). It is specifically consistent with the study area was rich in non-ferrous metal mines. In addition, a previous study reported that cadmium from mining sources was easily converted into the activated status and further enriched by rice^51^. Especially in acidic soil, the activated cadmium affected by mining could account for 40–60%^52^. Interestingly, the performance of the cadmium transport in soil from certain sources was not consistent with that of enrichment by rice, such as the traffic sources. As shown in Fig. 5b, cadmium from traffic sources could easily be enriched by rice, rather than transformed into an activated state. Other research suggested that the cadmium from traffic sources could increase the content of carbonate Cd in soil, resulting in the decrease of Cd_a_ proportion^53^. On the other side, leaf tissue also could increase the proportion of water-soluble Cd in plants^54^, indicating that cadmium in rice might not transfer from the soil directly. Furthermore, the contribution ratio of soil cadmium from animal husbandry sources was slightly lower than that from traffic sources (21.9 vs. 30.1%). However, the contribution ratios of Cd_a_ and Cd_r_ were 1.74 and 8.76%, respectively, indicating the migration ability of animal husbandry sources. Compared with the above three sources, the contribution ratio of cadmium from the other two sources (farming sources and natural sources) in the soil can be almost ignored.

### Priority source of Cd pollution

**Figure 6:**
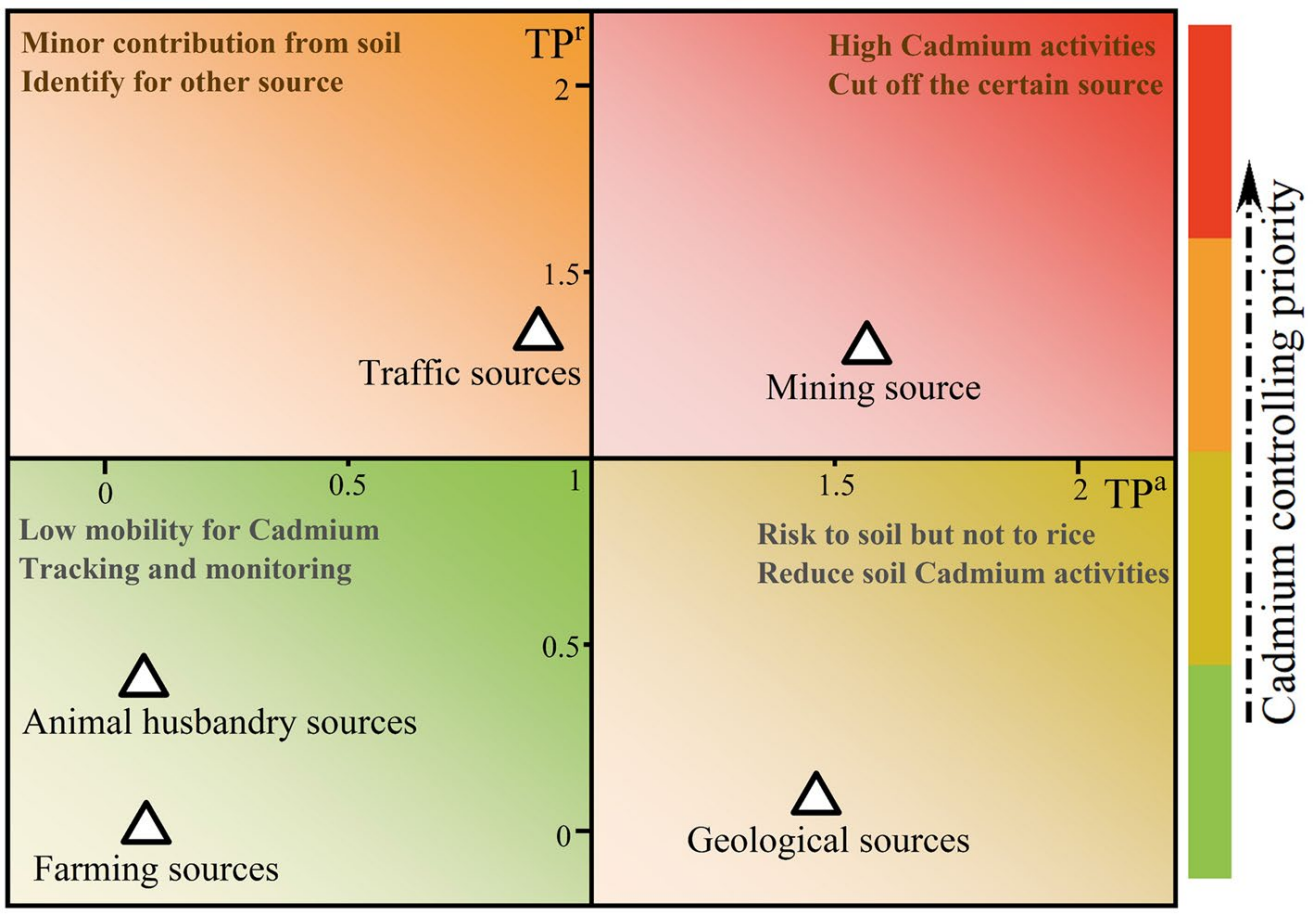
The priority of different sources on cadmium control.

As aforementioned, activated and enriched capacities of soil cadmium from different sources is unequal, suggesting that effective and priority cadmium control measures should target specific pollution sources. To reveal the priority sources for mitigating Cd pollution, we defined transfer potential (TP) to describe the ability of Cd from soil to rice (TP^r^ = Cd_r_/Cd_s_) and activated status (TP^a^ = Cd_a_/Cd_s_), respectively (Fig. 6). High levels of TP^r^ shows the strong cadmium activities which are easily enriched by rice highlighting the priority for cutting off the corresponding pollution sources. The value of TP^a^ illustrates the ability of cadmium to transform into an activated state, showing the risk to the soil rather than the rice. Hence, both of these two ratios should synthetically be considered for Cd mitigation in the soil–rice system. As shown in Fig. 6, the mining source has high values of both TP^r^ and TP^a^, which has the highest priority level of control. Our results are in line with a previous study^6,14^, that industrial activities posed a higher risk both to ecological and human health. Followed by traffic sources with the high-levels of TP^r^. Then the next is geological sources that were easy to be transformed into an activated state, and finally, animal husbandry sources and farming sources that only contributed to the total amount of soil cadmium.

### The spatial distribution of Cd contribution

**Figure 7:**
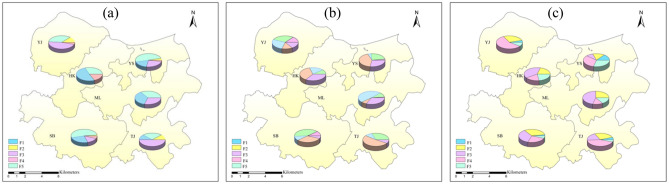
The distribution characteristics of Cd source contribution ratios in different towns, (**a**) soil cadmium; (**b**) activated cadmium; (**c**) rice cadmium.

As aforementioned, five factors of soil cadmium activated cadmium, and rice cadmium in the soil–rice system is based on the whole datasets of Xiangtan County. However, each town in Xiangtan County has a different industrial layout and different mineral resources^27^. To control the source of Cd pollution effectively, we analyzed the spatial distribution of Cd contribution in different towns. Figure 7 showed significant spatial heterogeneity in soil Cd distribution across the regional scale: cadmium pollution sources of any given type (soil cadmium, activated cadmium, and rice cadmium) are unequally distributed across locations. Associated underlying reasons are the disparities in the transport performance of Cd from the same source between the certain town and the overall study region. Roughly, the cadmium from all pollution sources, except for the farming sources, showed similar performances to that of the whole study regions in two-thirds of the towns. However, there were still some discrepancies between some towns with specific sources and the whole study region. For example, the soil cadmium from animal husbandry sources in YS town was easily transformed into an activated state and enriched by rice, which was contrary compared to the whole region. A possible explanation is that different types of livestock manure have various organic matters, especially solubility and acidity^55^. The use of livestock manure could bring in a large number of soil soluble substances, which had a small molecular weight and contained a large number of active substances such as carboxyl and hydroxyl groups, promoting the increase of soluble Cd concentration^54^.

## Conclusions

In this study, we proposed a novel hybrid framework to reveal significant differences in the migration ability of cadmium from various sources, and then to reveal the priority of controlling Cd pollution sources in soil–rice systems based on a high-dimensional geographical database. Firstly, the industry types of enterprises and the estimated Cd input flux are summarized in the high-dimensional geographical database. Then, based on the soil property, Principal component analysis is used to reveal the pollution sources of cadmium in soil, activated state, and rice, including farming sources, traffic sources, animal husbandry sources, mining source, and geological sources. To quantify the contribution of different Cd pollution sources, the estimated Cd input flux combined with PCA results was applied to estimate the contributions by the MLR method. Thereinto, the mining source is the dominant contributor among the five sources, accounting for 50.3 ± 11.1%. The difference in Cd contribution ratios from various sources indicated the significant disparities in the transport capacity of cadmium in the soil–rice system. The unequal activated and enriched capacities of soil cadmium from different sources suggest that effective and priority cadmium control measures should target specific pollution sources. Finally, to reveal the priority sources for mitigating Cd pollution, we defined transfer potential (TP) to express the ability of Cd from soil to rice (TP^r^ = Cd_r_/Cd_s_) and activated status (TP^a^ = Cd_a_/Cd_s_). The mining source has both high levels of TP^r^ and TP^a^, which were more easily activated and enriched by rice that should be controlled priority. Followed by traffic sources with a higher value of TP^r^, showing the risk to rice rather than the soil. In addition, Cadmium contamination shows a significant spatial heterogeneity at the regional scale that we attribute to the difference in Cd transport performance in different sources. The experience gained in our study can help inform efforts to control cadmium contamination at a regional scale that is undergoing significant food safety risks.

## Supplementary Information

Supplementary Information.

## Data Availability

The datasets used and/or analyzed during the current study available from the corresponding author on reasonable request.

## References

[1] Zou M (2021). Cadmium pollution of soil–rice ecosystems in rice cultivation dominated regions in China: A review. Environ. Pollut..

[2] Chen H (2018). Dietary cadmium intake from rice and vegetables and potential health risk: A case study in Xiangtan, southern China. Sci. Total Environ..

[3] Yang S (2019). An integrated analysis on source-exposure risk of heavy metals in agricultural soils near intense electronic waste recycling activities. Environ. Int..

[4] Liu P (2020). Accumulation and ecological risk of heavy metals in soils along the coastal areas of the Bohai Sea and the Yellow Sea: A comparative study of China and South Korea. Environ. Int..

[5] He M (2019). Ten-year regional monitoring of soil–rice grain contamination by heavy metals with implications for target remediation and food safety. Environ. Pollut..

[6] Guo G, Wang Y, Zhang D, Lei M (2021). Source-specific ecological and health risks of potentially toxic elements in agricultural soils in Southern Yunnan Province and associated uncertainty analysis. J. Hazard. Mater..

[7] Ali W (2020). Comprehensive review of the basic chemical behaviours, sources, processes, and endpoints of trace element contamination in paddy soil–rice systems in rice-growing countries. J. Hazard. Mater..

[8] Cao H (2010). Heavy metals in rice and garden vegetables and their potential health risks to inhabitants in the vicinity of an industrial zone in Jiangsu, China. J. Environ. Sci..

[9] Zhao D (2018). Coupling bioavailability and stable isotope ratio to discern dietary and non-dietary contribution of metal exposure to residents in mining-impacted areas. Environ. Int..

[10] Deng M (2021). Improving Cd risk managements of rice cropping system by integrating source-soil–rice-human chain for a typical intensive industrial and agricultural region. J. Clean. Prod..

[11] Lien K-W, Pan M-H, Ling M-P (2021). Levels of heavy metal cadmium in rice (*Oryza sativa* L.) produced in Taiwan and probabilistic risk assessment for the Taiwanese population. Environ. Sci. Pollut. Res..

[12] Arao T (2010). Heavy metal contamination of agricultural soil and countermeasures in Japan. Paddy Water Environ..

[13] Liu J, Qian M, Cai G, Yang J, Zhu Q (2007). Uptake and translocation of Cd in different rice cultivars and the relation with Cd accumulation in rice grain. J. Hazard. Mater..

[14] Hu Y, Cheng H, Tao S (2016). The challenges and solutions for cadmium-contaminated rice in China: A critical review. Environ. Int..

[15] Ahmed M, Eslamian M (2015). Laminar forced convection of a nanofluid in a microchannel: Effect of flow inertia and external forces on heat transfer and fluid flow characteristics. Appl. Therm. Eng..

[16] Gan Y (2019). Source quantification and potential risk of mercury, cadmium, arsenic, lead, and chromium in farmland soils of Yellow River Delta. J. Clean. Prod..

[17] Zhang J (2021). Source identification of Cd and Pb in typical farmland topsoil in the southwest of China: A case study. Sustainability.

[18] Wang J (2021). Source analysis of heavy metal pollution in agricultural soil irrigated with sewage in Wuqing, Tianjin. Sci. Rep..

[19] Wei C, Lei M, Chen T, Zhou C, Gu R (2022). Method on site-specific source apportionment of domestic soil pollution across China through public data mining: A case study on cadmium from non-ferrous industries. Environ. Pollut..

[20] Zhang H (2021). Risk sources quantitative appointment of ecological environment and human health in farmland soils: A case study on Jiuyuan District in China. Environ. Geochem. Health.

[21] Salim I (2019). Comparison of two receptor models PCA-MLR and PMF for source identification and apportionment of pollution carried by runoff from catchment and sub-watershed areas with mixed land cover in South Korea. Sci. Total Environ..

[22] Chen Z (2022). Combination of UNMIX, PMF model and Pb–Zn–Cu isotopic compositions for quantitative source apportionment of heavy metals in suburban agricultural soils. Ecotoxicol. Environ. Saf..

[23] Greger M (2008). Trace Elements as Contaminants and Nutrients: Consequences in Ecosystems and Human Health.

[24] Zhao K, Liu X, Xu J, Selim HM (2010). Heavy metal contaminations in a soil–rice system: Identification of spatial dependence in relation to soil properties of paddy fields. J. Hazard. Mater..

[25] Said I, Salman SA, Elnazer AA (2019). Multivariate statistics and contamination factor to identify trace elements pollution in soil around Gerga City, Egypt. Bull. Natl. Res. Centre.

[26] Li X, Geng T, Shen W, Zhang J, Zhou Y (2021). Quantifying the influencing factors and multi-factor interactions affecting cadmium accumulation in limestone-derived agricultural soil using random forest (RF) approach. Ecotoxicol. Environ. Saf..

[27] Wang X (2018). Quadratic discriminant analysis model for assessing the risk of cadmium pollution for paddy fields in a county in China. Environ. Pollut..

[28] MAPAPRC. Ministry of Agriculture and Rural Affairs of the People’s Republic of China (2000).

[29] Huang G (2022). A hybrid data-driven framework for diagnosing contributing factors for soil heavy metal contaminations using machine learning and spatial clustering analysis. J. Hazard. Mater..

[30] Thurston GD, Spengler JD (1985). A quantitative assessment of source contributions to inhalable particulate matter pollution in metropolitan Boston. Atmos. Environ..

[31] Li B, Zhang H, Zhang W, Li T (2021). The PCA-KD-KNN-based water chemistry identification model of water inrush source type in mine and its application. Arab. J. Geosci..

[32] Sakizadeh M, Zhang C (2021). Source identification and contribution of land uses to the observed values of heavy metals in soil samples of the border between the Northern Ireland and Republic of Ireland by receptor models and redundancy analysis. Geoderma.

[33] Yang Y, Yang X, He M, Christakos G (2020). Beyond mere pollution source identification: Determination of land covers emitting soil heavy metals by combining PCA/APCS, GeoDetector and GIS analysis. CATENA.

[34] Sanchez JM (2020). Linear calibrations in chromatography: The incorrect use of ordinary least squares for determinations at low levels, and the need to redefine the limit of quantification with this regression model. J. Sep. Sci..

[35] Li Z, Ma Z, van der Kuijp TJ, Yuan Z, Huang L (2014). A review of soil heavy metal pollution from mines in China: Pollution and health risk assessment. Sci. Total Environ..

[36] Zhang H (2011). Predicting As, Cd and Pb uptake by rice and vegetables using field data from China. J. Environ. Sci..

[37] Park HJ (2021). Cadmium phytoavailability from 1976 through 2016: Changes in soil amended with phosphate fertilizer and compost. Sci. Total Environ..

[38] Cui S-F (2021). Transfer characteristic of fluorine from atmospheric dry deposition, fertilizers, pesticides, and phosphogypsum into soil. Chemosphere.

[39] Huang Y (2015). An integrated approach to assess heavy metal source apportionment in peri-urban agricultural soils. J. Hazard. Mater..

[40] Zhang J, Hua P, Krebs P (2017). Influences of land use and antecedent dry-weather period on pollution level and ecological risk of heavy metals in road-deposited sediment. Environ. Pollut..

[41] Krailertrattanachai N, Ketrot D, Wisawapipat W (2019). The distribution of trace metals in roadside agricultural soils, Thailand. Handl. Skewed Data Comp. Two Popul. Methods.

[42] Wang S (2019). Spatial distribution and source apportionment of heavy metals in soil from a typical county-level city of Guangdong Province, China. Sci. Total Environ..

[43] Wang L, Gao S, Yin X, Yu X, Luan L (2019). Arsenic accumulation, distribution and source analysis of rice in a typical growing area in north China. Ecotoxicol. Environ. Saf..

[44] Li H (2020). Input of Cd from agriculture phosphate fertilizer application in China during 2006–2016. Sci. Total Environ..

[45] Rai PK, Lee SS, Zhang M, Tsang YF, Kim K-H (2019). Heavy metals in food crops: Health risks, fate, mechanisms, and management. Environ. Int..

[46] Shen B (2020). The optimum pH and Eh for simultaneously minimizing bioavailable cadmium and arsenic contents in soils under the organic fertilizer application. Sci. Total Environ..

[47] Du B (2020). Environmental and human health risks from cadmium exposure near an active lead–zinc mine and a copper smelter, China. Sci. Total Environ..

[48] Jiang H-H (2020). An integrated approach to quantifying ecological and human health risks from different sources of soil heavy metals. Sci. Total Environ..

[49] Qin G (2021). Soil heavy metal pollution and food safety in China: Effects, sources and removing technology. Chemosphere.

[50] Song T (2020). The origin of soil selenium in a typical agricultural area in Hamatong River Basin, Sanjiang Plain, China. CATENA.

[51] Cai L-M (2019). Heavy metal contamination and health risk assessment for children near a large Cu-smelter in central China. Sci. Total Environ..

[52] He B (2021). Exploring the fate of heavy metals from mining and smelting activities in soil-crop system in Baiyin, NW China. Ecotoxicol. Environ. Saf..

[53] Ben Seghier T, Bouhadjera K (2020). Pollution assessment of heavy metals in roadside agricultural soils. Pol. J. Environ. Stud..

[54] Liu J (2020). Variation of soil dissolved organic carbon under long-term different fertilizations and its correlation with maize yields. J. Soils Sediments.

[55] Meng T, Liu J (2021). Effects of bio-organic fertilizers on soil organic carbon components and biomass of shamrock. Bangladesh J. Bot..

