# Supplementary material for: The high-dimensional geographic dataset revealed significant differences in the migration ability of cadmium from various sources in paddy fields
Source: Sci Rep. 2023 Jan 28;13:1589. doi: 10.1038/s41598-023-28812-9 (PMC9884224; doi:10.1038/s41598-023-28812-9)
Supplement: Supplementary file 1 — Supplementary Information. [file 41598_2023_28812_MOESM1_ESM.docx]

**Supplementary Information for**

**The high-dimensional geographic dataset revealed significant differences in the migration ability of cadmium from various sources in paddy fields**

Feng Wang^a, b#^, Yanqiu Zhang^a, c#^, Ting Wu^d^, Lina Wu^a^, Guoliang Shi^b^, Yi An^a,*^

^a^ Agro-Environmental Protection Institute, Ministry of Agriculture and Rural Affairs, Tianjin 300071, China;

^b^ College of Environmental Science and Engineering, Nankai University, Tianjin 300350, China;

^c^ College of Resource and Environment, Huazhong Agricultural University, Wuhan 430070, China;

^d^ State Key Laboratory on Odor Pollution Control, Tianjin Academy of Eco-Environmental Sciences, Tianjin, 300191, China.

^#^These authors contributed equally to this paper.

^*^Corresponding author

Yi An. Email: simon8601@126.com

Table S1 The monitoring indexes and their corresponding detection methods

| Number | Element | Detection method | Standard |
| --- | --- | --- | --- |
| 1 | Cd_r_ | Atomic-Absorption Spectrophotometry | GB/T5009.15-2014 |
| 2 | Cd_a_ | CaCl_2_ extract | DB35/T 860-2008 |
| 3 | Cd_s_ | Graphite Furnace Atomic Absorption Spectrometry | GB/T17141-1997 |
| 4 | Pb | Graphite Furnace Atomic Absorption Spectrometry | GB/T17141-1997 |
| 5 | Cr | Flame Atomic Absorption Spectroscopy | NY/T 1121.12-2006 |
| 6 | Cu | Flame Atomic Absorption Spectroscopy | NY/T 296-1995 |
| 7 | Zn | Flame Atomic Absorption Spectroscopy | GB/T17138-1997 |
| 8 | Hg | Atomic Fluorescence Spectrometry | NY/T 395-2000 |
| 9 | As | Atomic Fluorescence Spectrometry | NY/T 395-2000 |
| 10 | K | Flame Aatomic Absorption Spectroscopy | NY/T 395-2000 |
| 11 | P | Atomic Absorption Spectrophotometry | NY/T 395-2000 |
| 12 | N | Kjeldahl method | HJ 717-2014 |
| 13 | Si | Acid immersion method | LY/T 1266-1999 |
| 14 | Se | Atomic Fluorescence method | NY/T 395-2000 |
| 15 | Na | Flame Atomic Absorption Spectroscopy | NY/T 890-2004 |
| 16 | Mo | Acid immersion method | LY/T 1259-1999 |
| 17 | Mn | Atomic Absorption Spectrophotometry | NY/T 890-2004 |
| 18 | Mg | Atomic Absorption Spectrophotometry | NY/T1121.13-2006 |
| 19 | Fe | Atomic Absorption Spectrophotometry | NY/T 890-2004 |
| 20 | Ca | Atomic Absorption Spectrophotometry | NY/T 1121.13-2006 |
| 21 | Al | Atomic Absorption Spectrophotometry | NY/T 890-2004 |
| 22 | Ni | Flame Atomic Absorption Spectroscopy | NY/T 1121.12-2006 |
| 23 | Ti | X Ray Fluorescence spectrometry | HJ 780-2015 |

Table S2 The monitoring results of soil properties in the study area

| Element | Contents(mg/kg) | Element | Contents(mg/kg) | Element | Contents(mg/kg) |
| --- | --- | --- | --- | --- | --- |
| Pb | 50.82±18.66 | Cr | 72.56±18.65 | Cu | 24.53±4.88 |
| Zn | 88.70±17.39 | Hg | 0.18±0.11 | As | 19.01±9.52 |
| K | 86.60±20.12 | P | 22.72±16.70 | N | 176.64±16.17 |
| Si | 261.05±10.89 | Se | 0.68±0.16 | Na | 1300.44±265.34 |
| Mo | 0.98±0.15 | Mn | 315.22±79.08 | Mg | 248.62±78.99 |
| Fe | 24.10±2.51 | Ca | 1247.51±881.95 | Al | 9.95±1.38 |
| Ni | 26.76±2.56 | Ti | 4.67±0.21 |  |  |

^*^ The results of soil element contents were shown as “average±standard deviation”

Table S3 The Kriging interpolation methods and their key parameters for each heavy metal.

|  | Kriging interpolation method | Model | Nugget value | Range value | Sill value | Nugget/Sill ratio | Standard mean error | Standard root mean square error |
| --- | --- | --- | --- | --- | --- | --- | --- | --- |
| Cd | Simple Kriging | Exponential model | 0.2420 | 0.1284 | 0.9869 | 24.5212 | -0.0561 | 1.2733 |
| Pb | Common kriging | Stable model | 216.3416 | 0.0679 | 341.7516 | 63.3038 | 0.0005 | 0.8470 |
| As | Simple Kriging | Stable model | 0.0001 | 0.0612 | 1.0113 | 0.0099 | 0.0116 | 0.9077 |
| Cr | Simple Kriging | Exponential model | 0.1336 | 0.0479 | 0.9998 | 13.3627 | -0.0055 | 1.0069 |
| Hg | Simple Kriging | Stable model | 0.3694 | 0.0730 | 0.9998 | 36.9474 | -0.0388 | 1.3389 |
| Zn | Common kriging | Stable model | 0.0779 | 0.0676 | 376.8283 | 0.0207 | 0.0019 | 1.1568 |
| Cu | Simple Kriging | Stable model | 0.1289 | 0.0766 | 1.0053 | 12.8220 | 0.0395 | 0.5261 |
| K | Common kriging | Rational quadratic equation model | 21.7981 | 0.3052 | 584.7549 | 3.7277 | 0.0269 | 0.7618 |
| P | Simple Kriging | Exponential model | 0.0001 | 0.0967 | 1.1518 | 0.0087 | 0.1352 | 0.5704 |
| N | Simple Kriging | Stable model | 0.0893 | 0.0823 | 1.0000 | 8.9300 | -0.0137 | 0.9608 |
| Si | Common kriging | Rational quadratic equation model | 0.1449 | 0.1506 | 145.0318 | 0.0999 | -0.0071 | 1.2030 |
| Se | Common kriging | Rational quadratic equation model | 0.0001 | 0.4149 | 0.0439 | 0.2278 | -0.0028 | 0.8277 |
| Na | Simple Kriging | Rational quadratic equation model | 0.0011 | 0.0855 | 1.1490 | 0.0957 | 0.0097 | 1.0659 |
| Mo | Simple Kriging | Stable model | 0.0012 | 0.0844 | 1.2393 | 0.0968 | -0.0009 | 1.2459 |
| Mn | Simple Kriging | Stable model | 0.0011 | 0.0681 | 1.0798 | 0.1019 | 0.0185 | 1.4042 |
| Mg | Simple Kriging | Rational quadratic equation model | 0.0011 | 0.1312 | 1.1957 | 0.0920 | -0.0040 | 1.1102 |
| Fe | Common kriging | Stable model | 0.0001 | 0.3476 | 13.9267 | 0.0007 | 0.0073 | 0.6994 |
| Ca | Simple Kriging | Rational quadratic equation model | 0.0011 | 0.1374 | 1.1294 | 0.0974 | 0.0055 | 1.2390 |
| Al | Simple Kriging | Stable model | 0.0012 | 0.0908 | 1.2316 | 0.0974 | 0.0295 | 1.4103 |
| Ni | Simple Kriging | Stable model | 0.1915 | 0.1374 | 0.9296 | 20.6003 | 0.0166 | 0.6423 |
| Ti | Common kriging | Exponential model | 0.0001 | 0.0052 | 0.0003 | 33.3333 | -0.0224 | 1.3200 |


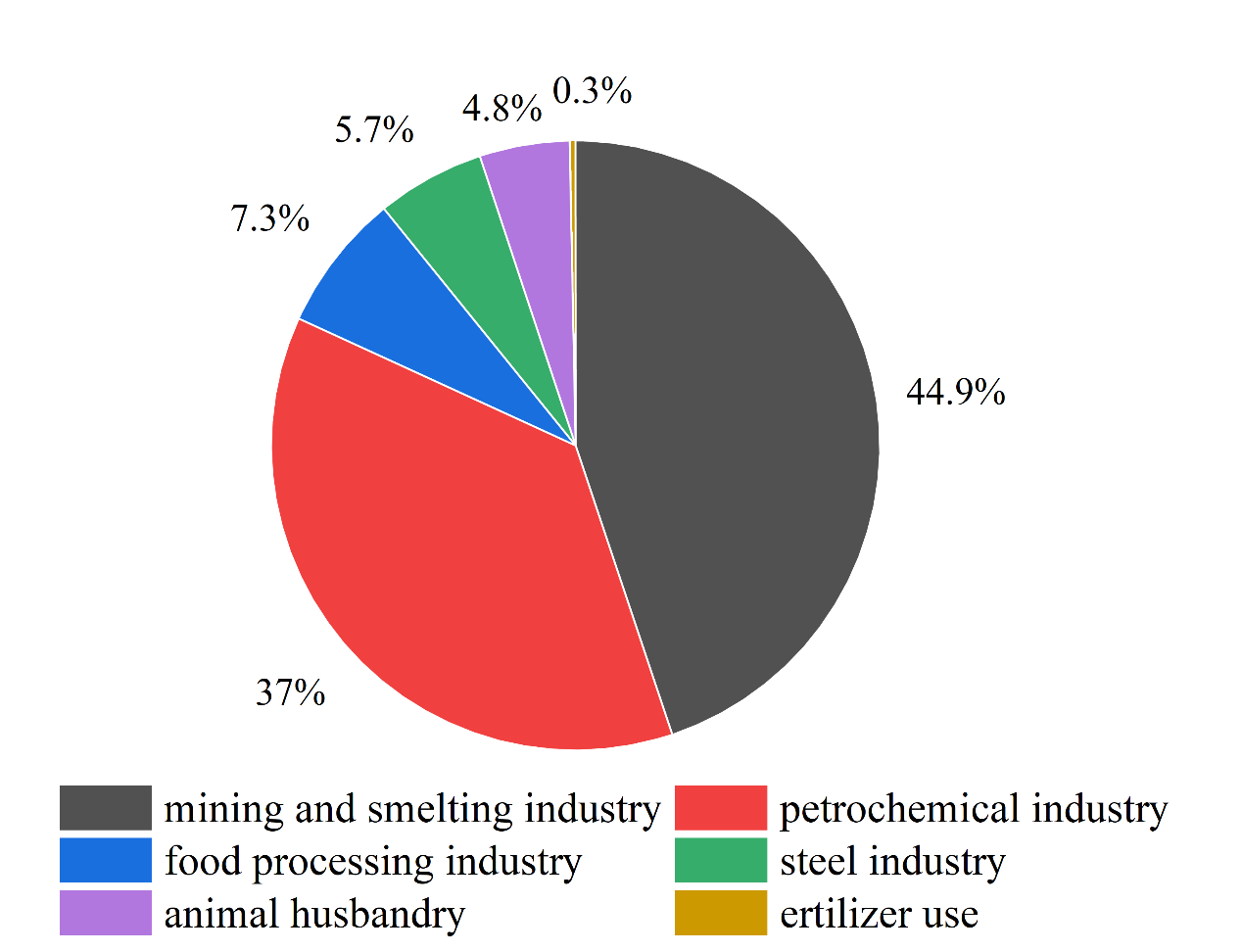


**Fig.S1** The proportion of the calculation of Cd input flux in the study area.
